# Assessing the predictive value of time-in-range level for the risk of postoperative infection in patients with type 2 diabetes: a cohort study

**DOI:** 10.3389/fendo.2025.1539039

**Published:** 2025-04-15

**Authors:** Ying Wu, Rui Xv, Qinyun Chen, Ranran Zhang, Min Li, Chen Shao, Guoxi Jin, Xiaolei Hu

**Affiliations:** ^1^ The Department of Endocrinology, The First Affiliated Hospital of Bengbu Medical University, Bengbu, Anhui, China; ^2^ The Department of Endocrinology, The Second Affiliated Hospital of Bengbu Medical University, Bengbu, Anhui, China; ^3^ The National Metabolic Management Center, The First Affiliated Hospital of Bengbu Medical University, Bengbu, Anhui, China

**Keywords:** type 2 diabetes, time in range, postoperative infection, risk factors, clinical prediction model

## Abstract

**Aim:**

To analyze the correlation between preoperative time-in-range (TIR) levels and postoperative infection in patients with type 2 diabetes mellitus (T2DM) and to evaluate the value of the TIR as a predictor of postoperative infection in patients with T2DM.

**Methods:**

A total of 656 patients with T2DM during the perioperative period were divided into a TIR standard group (TIR≥70%) and a TIR nonstandard group (TIR<70%) according to the TIR value. Modified Poisson regression was used to analyze postoperative risk factors in patients with T2DM. All patients were subsequently divided into a training set and a validation set at a ratio of 7:3. LASSO regression and the Boruta algorithm were used to screen out the predictive factors related to postoperative infection in T2DM patients in the training set. The discrimination and calibration of the model were evaluated by the area under the receiver operating characteristic curve (ROC) and calibration curve, and the clinical net benefit of the model was evaluated and verified through the decision analysis (DCA) curve. Finally, a forest plot was used for relevant subgroup analysis.

**Results:**

Modified Poisson regression analysis revealed that the TIR was a risk factor for postoperative infection in T2DM patients, and when the TIR was <70%, the risk of postoperative infection increased by 52.2% (P <0.05). LASSO regression and Boruta algorithm screening variables revealed that the TIR, lymphocytes, neutrophils, total serum cholesterol, superoxide dismutase and type of incision were predictive factors for postoperative infection in patients with T2DM (P<0.05). The calibration curve confirmed that the model predictions were consistent with reality, and the decision curve confirmed that the model had better clinical benefits. Finally, the results of the subgroup analysis revealed that in each subgroup, the risk of postoperative infection was greater when the TIR was <70% than when the TIR was ≥70%, and there was no interaction between subgroups.

**Conclusion:**

The TIR is related to postoperative infection and can be used as a new indicator to predict the risk of postoperative infection in patients with type 2 diabetes mellitus.

## 1 Introduction

Diabetes is a common disease in perioperative patients, and nearly half of patients with type 2 diabetes mellitus (T2DM) require surgery in their lifetime (1). Studies have shown that adverse events such as surgical site infection, secondary surgical intervention, and death are more common in diabetic patients than in nondiabetic patients (2) and that good perioperative blood glucose control can reduce the occurrence of these adverse events (3). Glycated hemoglobin (HbA1c), a standard blood glucose control measure, reflects the blood glucose level in the past 2–3 months, but it cannot reflect real-time blood glucose changes and is easily affected by factors such as hemoglobin and pregnancy (4). The pain caused by frequent fingertip blood glucose measurements reduces patient compliance. In recent years, with the rise of continuous glucose monitoring (CGM) instruments, CGM has been widely used in clinical practice and has successfully solved the above problems. The time-in-range (TIR), a derived index of CGM, has been widely used to evaluate the level of glycemic control (5). TIR generally refers to the time or percentage of glucose within the target range (usually 3.9~10.0 mmol/L) within 24 hours.

As a clinical tool that can predict the risk of outcome, prediction models have been mostly mentioned in recent years for postoperative infections in cancer or nondiabetic patients. However, infection, a common postoperative complication in diabetic patients, has received little attention. During clinical practice, we found that T2DM patients with higher TIR levels had fewer postoperative infections. For further verification, we designed this study to explore the relationship between preoperative TIR levels and postoperative infections in patients with perioperative T2DM. Moreover, whether the TIR can be used as a new indicator to predict the risk of postoperative infection in patients with T2DM is unknown.

## 2 Research population and research methods

**Figure 1:**
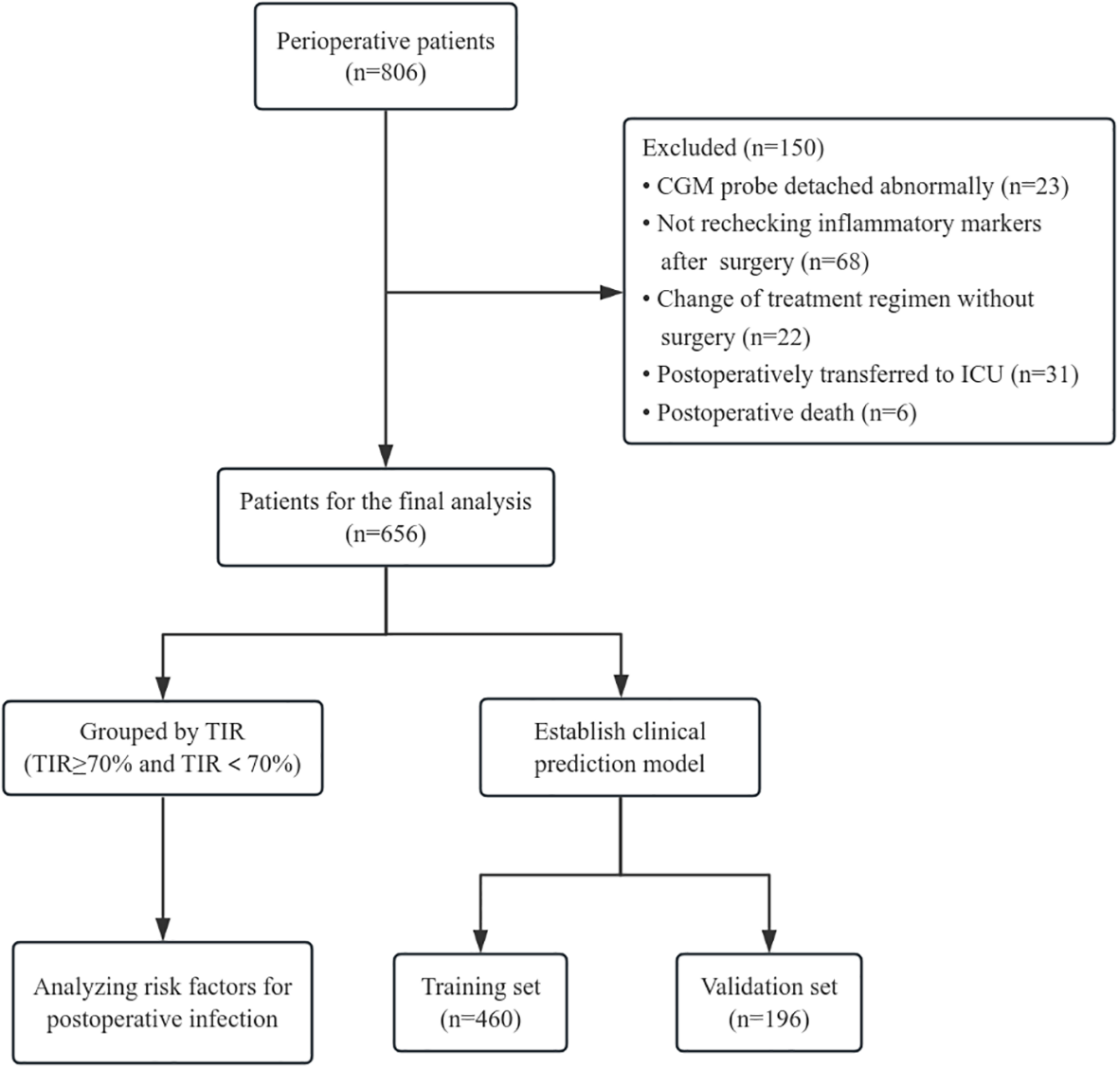
Diagram of the steps.

We included 806 patients with type 2 diabetes in the perioperative period from September 1, 2022, to July 31, 2024, at the First Affiliated Hospital of Bengbu Medical University, all of whom met the criteria for the diagnosis and classification of *Guideline for the Prevention and Treatment of Type 2 Diabetes Mellitus in China (2020 edition)* (6). After admission, the patients uniformly donned a CGM (product model: FreeStyle Libre; manufacturer: Abbott Diabetes Care UK) to monitor blood glucose, and a professional in-hospital blood glucose management team conducted blood glucose control on the basis of blood glucose conditions. During the observation period, there were 23 cases of abnormal damage and detachment of CGM probes, 68 cases of inflammation indicators were not recorded after surgery, 31 cases were transferred to the intensive care unit (ICU) ward after surgery, 22 cases changed the treatment plan without surgery, and 6 cases died. The final number of enrolled patients was 656. According to *International Consensus on Use of Continuous Glucose Monitoring* (7) and *the International Consensus on Time in Range* (8), the TIR of adults with type 1 or type 2 diabetes individuals should be greater than 70%. And Richard et al. have previously confirmed the recommendation of using TIR 70% as the baseline target in their study on the risk of adverse events associated with TIR in T2DM patients, and when TIR>70%, severe hypoglycemia and microvascular events are lower (9). Therefore, we chose TIR 70% as the cutoff point, and divided enrolled patients into two groups, TIR≥70% and TIR<70% as exposure factors, and postoperative infection during hospitalization as the research outcome. In addition, all patients were divided into training sets and validation sets at a ratio of 7:3 for analysis of the prediction model (**Figure 1**). The general information and relevant laboratory test data of all enrolled patients were collected. This study was approved by the Research Ethics Committee of the First Affiliated Hospital of Bengbu Medical University (approval number: 2022088).

### 2.1 Inclusion and discharge standards

The inclusion criteria were as follows: 1. Age ≥ 18 years old; 2. Patients with type 2 diabetes in the perioperative period who were wearing CGM for ≥ 3 days before surgery; 3. No antibiotics were used within one week before admission or before surgery, and preoperative white blood cell, neutrophil and C-reactive protein levels were within the normal range;

Exclusion criteria: 1. Patients who were infected or used antibacterial drugs before surgery; 2. Severely abnormal liver and kidney function, abnormal coagulation function, a severe lack of granulocytes and circulatory dysfunction; 3. An incision was made during the suppurative and acute inflammatory stages.

### 2.2 Clinical data collection

Basic patient information, including sex, age, duration of diabetes, previous medication, smoking history, drinking history, height, weight, body mass index (BMI), systolic blood pressure (SBP), and diastolic blood pressure (DBP), was collected upon admission. On admission, inflammatory indicators related to white blood cells (WBC), neutrophils (NEU), lymphocytes (LYM), C-reactive protein (CRP), alanine aminotransferase (ALT), aspartate aminotransferase (AST), total bilirubin (TB), albumin (ALB), creatinine (Cr), total serum cholesterol (TC), triglyceride (TG), high-density lipoprotein (HDL), low-density lipoprotein (LDL) and other related biochemical indicators were measured. Surgical data related to surgical methods, surgical grades and types of incisions were obtained. The CGM data were scanned and uploaded through the Gplus blood glucose management system to form a blood glucose map, and the time in range (TIR), time above range (TAR), time below range (TBR), coefficient of variation of blood glucose (CV), blood glucose standard deviation (SD), and mean amplitude of glycemic excursions (MAGE) were recorded. The average TIR of the patient 72 hours before surgery was calculated. According to *the Surgical Site Infection Guidelines* promulgated by the American College of Surgeons and the Infection Society in 2016 (10) and *The China Perioperative Infection Prevention and Management Guidelines* promulgated by China in 2023 (11), any of the following postoperative conditions are defined as postoperative infection: 1. Wound infection, including a) purulent drainage, b) positive bacterial culture results at the wound site, c) secondary human-induced opening of the incision, and d) a diagnosis of infection by a clinician; 2. Systemic infection: severe sepsis or sepsis (caused by wounds) or bacterial growth according to blood culture results; 3. White blood cell, neutrophil, and C-reactive protein indicators are all higher than the normal range. The following formula was used to calculate the serum composite index: (1) triglyceride-glucose (TyG) index = ln [fasting triglyceride (mg/dl) × fasting blood glucose (mg/dl)/2]; (2) high-stress blood glucose ratio (SHR) = fasting admission blood glucose (mg/dl)/[28.7×glycated hemoglobin (%)-46.7]; and (3) triglyceride/HDL ratio (THR) = fasting triglyceride (mmol/L)/HDL (mmol/L).

### 2.3 Statistical analysis

The data were statistically analyzed via SPSS software (version 26.0; SPSS Inc., Chicago, IL, USA). Normally distributed variables are expressed as the means ± standard deviations and were analyzed via t tests. Nonnormally distributed measurement data are expressed as the medians and interquartile range [M(Q1,Q2)] and were analyzed via the Mann–Whitney U test. Count data are expressed as the number of cases or percentages and were analyzed via the chi-square test. Modified Poisson regression was used to analyze the risk factors for postoperative infection in patients with T2DM, and the results are expressed as RR values. R4.3.3 software (http://www.r-project.org) and Python 3.12.0 software (http://www.python.org) were used, and the LASSO regression and Boruta algorithms were used to screen the relevant factors of postoperative infection in the training set. The intersection of the two was taken as the predictive factor, and a column chart was drawn. The area under the receiver operating characteristic (ROC) curve and calibration curve were used to evaluate the discrimination and calibration of the model, and decision curve analysis (DCA) was used to evaluate the clinical net benefit of the model and verify it. Logistic regression was used to analyze the risk ratio of postoperative infection in T2DM patients with TIR≥70% and TIR<70% in different subgroups, and a forest plot was drawn. P<0.05 indicated that the difference was statistically significant.

## 3 Results

### 3.1 Basic characteristics of the two groups: TIR≥70% and TIR<70%

As shown in **Table 1**, there were 281 patients in the TIR≥70% group and 375 patients in the TIR<70% group. The proportion of female patients, diabetes duration and postoperative infection rate in the TIR<70% group were significantly greater than those in the TIR≥70% group (*P*<0.05). The BMI, CHE, TBA, ALB, and THR of patients in the TIR≥70% group were significantly greater than those in the TIR<70% group (*P*<0.05). The levels of GGT, DB, and HDL in patients in the TIR≥70% group were significantly lower than those in patients in the TIR<70% group (*P*<0.05). The TAR, CV, SD, and MAGE of patients in the TIR≥70% group were significantly lower than those in the TIR<70% group (*P*<0.001). There was no significant difference in the other indicators between the two groups (*P*>0.05).

**Table 1:** Baseline comparison of clinical data between two groups of people.

Characteristic	Total (n = 656)	TIR<70% (n = 375)	TIR≥70% (n = 281)	t/z/χ²	*P*
Sex (%)				4.84	0.028*
Female	343 (52.29)	210 (56.00)	133 (47.33)		
Male	313 (47.71)	165 (44.00)	148 (52.67)		
Age (years)	60.95 ± 11.47	60.95 ± 11.71	60.95 ± 11.16	-0.01	0.995
Height (cm)	164.29 ± 8.66	164.61 ± 8.18	163.85 ± 9.25	1.12	0.264
Weight (kg)	66.63 ± 12.44	65.90 ± 11.70	67.61 ± 13.31	-1.74	0.082
BMI (kg/m^2^)	24.66 ± 3.94	24.25 ± 3.59	25.21 ± 4.30	-3.10	0.002*
SBP (mmHg)	137.40 ± 20.46	137.99 ± 20.11	136.61 ± 20.93	0.85	0.394
DBP (mmHg)	80.02 ± 12.41	79.67 ± 11.53	80.47 ± 13.50	-0.82	0.413
Smoking (%)				1.11	0.293
No	526 (80.18)	306 (81.60)	220 (78.29)		
Yes	130 (19.82)	69 (18.40)	61 (21.71)		
Drinking (%)				0.15	0.696
No	562 (85.67)	323 (86.13)	239 (85.05)		
Yes	94 (14.33)	52 (13.87)	42 (14.95)		
Duration (months)	120.00 (60.00,180.00)	120.00 (60.00,180.00)	112.00 (60.00,160.00)	-2.14	0.032*
Antidiabetic drugs (%)				5.11	0.164
Untreated	54 (8.23)	23 (6.13)	31 (11.03)		
Oral drugs	363 (55.34)	212 (56.53)	151 (53.74)		
Insulin	183 (27.90)	107 (28.53)	76 (27.05)		
Both	56 (8.54)	33 (8.80)	23 (8.19)		
GLU(mmol/L)	10.76 ± 4.49	10.84 ± 4.49	10.66 ± 4.50	0.50	0.614
HbA1c (%)	9.80 ± 2.00	9.90 ± 2.04	9.66 ± 1.94	1.48	0.140
WBC (10^9/L)	6.61 ± 1.64	6.65 ± 1.61	6.56 ± 1.67	0.72	0.473
NEU (10^9/L)	4.08(3.12,5.27)	4.15(3.23,5.32)	4.04(2.97,5.22)	-1.07	0.283
LYM (10^9/L)	1.73 ± 0.66	1.72 ± 0.66	1.74 ± 0.66	-0.53	0.595
CRP (mg/L)	4.00(2.00,5.98)	4.00(2.13,5.89)	4.00(1.80,6.30)	-0.06	0.953
HGB (g/L)	125.01 ± 21.85	124.17 ± 21.90	126.14 ± 21.78	-1.14	0.253
D-D (mg/L)	0.45(0.22,1.15)	0.46(0.23,1.15)	0.41(0.21,1.15)	-0.61	0.544
ALT (U/L)	16.00 (13.00,24.00)	17.00 (13.00,25.00)	16.00 (13.00, 23.00)	-0.27	0.786
AST (U/L)	18.00 (14.00, 26.00)	18.00 (14.00, 26.00)	17.00 (14.00, 26.00)	-0.38	0.707
ALP (U/L)	84.16 ± 34.67	85.91 ± 36.67	81.82 ± 31.72	1.53	0.127
CHE (U/L)	7687.81 ± 2332.70	7477.70 ± 2370.85	7968.20 ± 2254.61	-2.68	0.008*
GGT (U/L)	24.00 (16.00,39.25)	26.00 (16.50, 43.00)	22.00 (15.00, 35.00)	-2.48	0.013*
TB (µmol/L)	10.66 ± 5.62	10.57 ± 5.73	10.78 ± 5.47	-0.47	0.641
DB (µmol/L)	3.10 (2.20, 4.40)	3.20 (2.40, 4.80)	2.90 (2.10, 4.00)	-2.95	0.003*
IB (µmol/L)	6.90 ± 3.96	6.66 ± 4.02	7.22 ± 3.86	-1.81	0.071
TBA (µmol/L)	4.45 (2.60, 7.80)	4.20 (2.45, 7.20)	4.80 (2.70, 8.90)	-2.00	0.045*
ALB (g/L)	39.94 ± 5.03	39.41 ± 5.15	40.63 ± 4.78	-3.09	0.002*
UA (µmol/L)	266.08 ± 93.49	265.50 ± 97.61	266.86 ± 87.87	-0.18	0.853
Cr (µmol/L)	64.32 ± 20.82	64.91 ± 20.84	63.53 ± 20.80	0.84	0.402
BUN (mmol/L)	5.99 ± 2.50	6.10 ± 2.33	5.85 ± 2.71	1.28	0.201
HCO3¯ (mmol/L)	23.40 ± 3.84	23.31 ± 3.70	23.51 ± 4.02	-0.64	0.520
TC (mmol/L)	4.48 ± 1.50	4.46 ± 1.48	4.51 ± 1.52	-0.44	0.662
TG (mmol/L)	1.48 (1.04, 2.19)	1.51 (1.01, 2.15)	1.47 (1.08, 2.24)	-1.13	0.260
HDL (mmol/L)	1.07 ± 0.37	1.11 ± 0.38	1.02 ± 0.34	3.25	0.001*
LDL (mmol/L)	2.53 ± 1.05	2.51 ± 1.14	2.57 ± 0.90	-0.70	0.487
ApoA (mmol/L)	1.01 ± 0.32	1.01 ± 0.32	1.00 ± 0.31	0.32	0.747
ApoB (mmol/L)	0.82 ± 0.25	0.81 ± 0.24	0.83 ± 0.25	-1.07	0.287
CysC (mg/L)	1.07 ± 0.50	1.08 ± 0.52	1.05 ± 0.47	0.54	0.589
RBP (mg/L)	34.96 ± 15.22	34.62 ± 15.55	35.40 ± 14.79	-0.65	0.513
SOD (KU/L)	150.80 ± 41.17	149.19 ± 46.56	152.95 ± 32.56	-1.16	0.247
TAR (%)	25.50 (14.78, 38.52)	34.20 (24.60, 47.60)	15.80 (10.00, 23.10)	-14.46	<.001*
TBR (%)	1.90 (0.40, 5.82)	1.80 (0.40, 5.95)	2.10 (0.50, 5.80)	-0.70	0.483
CV (%)	35.26 ± 7.65	37.00 ± 7.87	32.94 ± 6.69	7.13	<.001*
SD (mmol/L)	2.99 ± 0.84	3.35 ± 0.81	2.51 ± 0.62	15.04	<.001*
MAGE (mmol/L)	7.09 ± 2.12	7.86 ± 2.15	6.06 ± 1.59	12.32	<.001*
SHR	0.85 ± 0.35	0.85 ± 0.36	0.85 ± 0.34	-0.03	0.977
TyG	3.64 ± 0.78	3.61 ± 0.76	3.67 ± 0.80	-1.08	0.281
THR	1.51 (0.97, 2.37)	1.47 (0.86, 2.18)	1.54 (1.02, 2.58)	-2.44	0.014*
Surgical methods (%)				0.52	0.473
Open	428 (65.24)	249 (66.40)	179 (63.70)		
Non-open	228 (34.76)	126 (33.60)	102 (36.30)		
Surgical grade (%)				-	0.213
1	1 (0.15)	0 (0.00)	1 (0.36)		
2	38 (5.79)	25 (6.67)	13 (4.63)		
3	230 (35.06)	138 (36.80)	92 (32.74)		
4	387 (58.99)	212 (56.53)	175 (62.28)		
Type of incision (%)				4.70	0.095
I	350 (53.35)	190 (50.67)	160 (56.94)		
II	259 (39.48)	152 (40.53)	107 (38.08)		
III	47 (7.16)	33 (8.80)	14 (4.98)		
Postoperative infection (%)				43.50	<.001*
No	227 (34.60)	90 (24.00)	137 (48.75)		
Yes	429 (65.40)	285 (76.00)	144 (51.25)		

BMI, body mass index; SBP, systolic blood pressure; DBP, diastolic blood pressure; HbA1c, Glycated hemoglobin; WBC, white blood cell; NEU, neutrophil, LYM, lymphocyte; CRP, C-reactive protein; ALT, alanine aminotransferase; AST, aspartate aminotransferase; ALB, albumin; Cr, creatinine; TC, total serum cholesterol; TG, triglyceride; HDL, high-density lipoprotein; LDL, low-density lipoprotein; Glu, glucose; HbA1c, glycosylated hemoglobin; HGB, hemoglobin; D-D, D-dimer; GGT, γGlutamine Transferase; ALP, alkaline phosphatase; CHE, cholinesterase; TB, total bilirubin; DB, Direct bilirubin; IB, Indirect bilirubin; TBA, Total bile acids; UA, uric acid; BUN, Blood urea nitrogen; ApoA, Apolipoprotein A; ApoB, Apolipoprotein B; CysC, Cystatin C; RBP, Retinol binding protein; SOD, Superoxide Dismutase; TIR, time in range; TAR, time above range; TBR, time below range; CV, coefficient of variation of blood glucose; SD, blood glucose standard deviation; MAGE, mean amplitude of glycemic excursions; TyG, triglyceride-glucose; SHR, high-stress blood glucose ratio; THR, triglyceride/HDL ratio. * p<0.05.

### 3.2 Modified poison regression analysis of related risk factors

To identify the risk factors related to postoperative infection in patients with T2DM, we used whether postoperative infection occurred as the dependent variable and other factors as independent variables through modified Poisson regression analysis. As shown in **Table 2**, sex, surgical grade, type of incision, TIR, SBP, ALT, ALP, GGT, IB, CysC, RBP, SD, and MAGE are risk factors for postoperative infection in T2DM patients. The risk of postoperative infection increased by 52.2% when the TIR was <70% compared when the TIR was ≥70% (*p*<0.001).

**Table 2:** Analysis of risk factors by modified Poisson regression.

Characteristic	B	*p*	RR	95% CI	
				lower limit	upper limit
Sex					
Male	0^a^	0.002	1.232	1.077	1.409
Female	0.209		1		
Smoking					
No	0.010	0.892	1.010	0.879	1.160
Yes	0^a^		1		
Drinking					
No	-0.017	0.822	0.984	0.851	1.136
Yes	0^a^		1		
Antidiabetic drugs					
Untreated	0.023	0.866	1.023	0.787	1.329
Oral drugs	-0.053	0.601	0.948	0.777	1.157
Insulin	0.011	0.916	1.011	0.827	1.236
Both	0^a^		1		
Surgical methods					
Open	0.008	0.900	1.008	0.895	1.134
Non-open	0^a^		1		
Surgical grade					
1	0.215	0.331	1.240	0.804	1.912
2	-0.344	0.015	0.709	0.537	0.935
3	-0.336	<0.001	0.715	0.621	0.822
4	0^a^		1		
Type of incision					
I	-0.492	<0.001	0.612	0.514	0.727
II	-0.245	0.008	0.783	0.654	0.938
III	0^a^		1		
TIR					
<70%	0.420	<0.001	1.522	1.314	1.763
≥70%	0^a^		1		
Age (years)	0.002	0.531	1.002	0.996	1.007
BMI (kg/m^2^)	0.009	0.309	1.009	0.992	1.025
SBP (mmHg)	-0.005	0.005	0.995	0.991	0.998
DBP (mmHg)	0.003	0.344	1.003	0.997	1.009
Duration (months)	7.354E-05	0.829	1.000	0.999	1.001
HbA1c (%)	-0.008	0.787	0.992	0.935	1.052
WBC (10^9/L)	0.014	0.801	1.014	0.907	1.135
NEU (10^9/L)	0.090	0.176	1.094	0.960	1.247
LYM (10^9/L)	-0.014	0.841	0.986	0.861	1.130
HGB (g/L)	-0.003	0.093	0.997	0.994	1.000
CRP (mg/L)	-0.003	0.776	0.997	0.976	1.018
D-D (mg/L)	-0.003	0.918	0.997	0.949	1.049
ALT (U/L)	-0.006	0.014	0.994	0.989	0.999
AST (U/L)	0.001	0.684	1.001	0.995	1.007
ALP (U/L)	-0.002	0.007	0.998	0.996	0.999
GGT (U/L)	0.002	0.008	1.002	1.001	1.004
CHE (U/L)	2.110E-06	0.908	1.000	1.000	1.000
DB (µmol/L)	0.008	0.345	1.008	0.991	1.026
IB (µmol/L)	0.013	0.047	1.013	1.000	1.027
TBA (µmol/L)	0.005	0.350	1.005	0.995	1.015
ALB (g/L)	0.012	0.122	1.012	0.997	1.027
Glu (mmol/L)	-0.007	0.782	0.993	0.948	1.041
UA (µmol/L)	0.000	0.609	1.000	0.999	1.000
Cr (µmol/L)	0.001	0.666	1.001	0.997	1.004
BUN (mmol/L)	-0.007	0.607	0.993	0.967	1.020
HCO3- (mmol/L)	0.006	0.443	1.006	0.991	1.020
TC (mmol/L)	-0.042	0.356	0.959	0.878	1.048
TG (mmol/L)	-0.131	0.063	0.877	0.764	1.007
HDL (mmol/L)	-0.036	0.742	0.965	0.781	1.192
LDL (mmol/L)	0.049	0.260	1.050	0.964	1.144
ApoA (mmol/L)	0.138	0.300	1.148	0.884	1.490
ApoB (mmol/L)	-0.340	0.079	0.712	0.487	1.041
CysC (mg/L)	0.175	0.002	1.191	1.064	1.333
RBP (mg/L)	-0.005	0.029	0.995	0.990	0.999
SOD (KU/L)	-0.001	0.214	0.999	0.996	1.001
TAR (%)	-0.006	0.075	0.994	0.987	1.001
TBR (%)	0.007	0.259	1.007	0.995	1.020
CV (%)	-0.022	0.079	0.979	0.955	1.003
SD (mmol/L)	0.301	0.015	1.352	1.061	1.722
MAGE (mmol/L)	-0.050	0.038	0.951	0.907	0.997
TyG	0.179	0.149	1.196	0.938	1.525
SHR	0.025	0.927	1.025	0.602	1.746
THR	0.026	0.079	1.026	0.997	1.056

BMI, body mass index; SBP, systolic blood pressure; DBP, diastolic blood pressure; HbA1c, Glycated hemoglobin; WBC, white blood cell; NEU, neutrophil, LYM, lymphocyte; CRP, C-reactive protein; ALT, alanine aminotransferase; AST, aspartate aminotransferase; ALB, albumin; Cr, creatinine; TC, total serum cholesterol; TG, triglyceride; HDL, high-density lipoprotein; LDL, low-density lipoprotein; Glu, glucose; HbA1c, glycosylated hemoglobin; HGB, hemoglobin; D-D, D-dimer; GGT, γGlutamine Transferase; ALP, alkaline phosphatase; CHE, cholinesterase; TB, total bilirubin; DB, Direct bilirubin; IB, Indirect bilirubin; TBA, Total bile acids; UA, uric acid; BUN, Blood urea nitrogen; ApoA, Apolipoprotein A; ApoB, Apolipoprotein B; CysC, Cystatin C; RBP, Retinol binding protein; SOD, Superoxide Dismutase; TIR, time in range; TAR, time above range; TBR, time below range; CV, coefficient of variation of blood glucose; SD, blood glucose standard deviation; MAGE, mean amplitude of glycemic excursions; TyG, triglyceride-glucose; SHR, high-stress blood glucose ratio; THR, triglyceride/HDL ratio. parameter "a": Set to zero due to this parameter redundancy.

### 3.3 Basic characteristics of the training set and validation set

To further clarify whether the TIR is a predictive factor for postoperative infection in patients with T2DM, we constructed a clinical prediction model. First, all enrolled patients were divided into a training set and a validation set at a ratio of 7:3. The results revealed that there was no significant difference in the basic characteristics of the two groups (**Table 3**).

**Table 3:** Comparison of baseline features between training group and validation group.

Characteristic	Test (n=196)	Train (n=460)	p.overall
Sex			0.395
Male	97 (49.5%)	246 (53.5%)	
Female	99 (50.5%)	214 (46.5%)	
Age (years)	61.9 (10.7)	60.5 (11.8)	0.131
Height (cm)	163 (9.25)	165 (8.37)	0.121
Weight (kg)	66.0 (11.7)	66.9 (12.8)	0.407
BMI (kg/m^2^)	24.8 (3.97)	24.6 (3.93)	0.576
SBP (mmHg)	137 (20.6)	138 (20.4)	0.785
DBP (mmHg)	79.9 (12.3)	80.1 (12.5)	0.852
Smoking			0.475
No	161 (82.1%)	365 (79.3%)	
Yes	35 (17.9%)	95 (20.7%)	
Drinking			0.887
No	169 (86.2%)	393 (85.4%)	
Yes	27 (13.8%)	67 (14.6%)	
Duration (months)	133 (82.9)	120 (80.6)	0.064
Antidiabetic drugs (%)			0.255
Untreated	10 (5.10%)	44 (9.57%)	
Oral drugs	109 (55.6%)	254 (55.2%)	
Insulin	58 (29.6%)	125 (27.2%)	
Both	19 (9.69%)	37 (8.04%)	
Surgery methods			0.063
Open	117 (59.7%)	311 (67.6%)	
Non-open	79 (40.3%)	149 (32.4%)	
Surgical grade (%)			0.173
1	0 (0.00%)	1 (0.22%)	
2	6 (3.06%)	32 (6.96%)	
3	68 (34.7%)	162 (35.2%)	
4	122 (62.2%)	265 (57.6%)	
Type of incision (%)			0.127
I	106 (54.1%)	244 (53.0%)	
II	82 (41.8%)	177 (38.5%)	
III	8 (4.08%)	39 (8.48%)	
HbA1c (%)	9.74 (1.94)	9.82 (2.03)	0.615
WBC (10^9/L)	6.71 (1.63)	6.57 (1.64)	0.323
NEU (10^9/L)	4.24 (1.38)	4.18 (1.38)	0.614
LYM (10^9/L)	1.76 (0.67)	1.72 (0.66)	0.488
CRP (mg/L)	4.19 (2.62)	4.15 (2.63)	0.854
HGB (g/L)	124 (22.7)	125 (21.5)	0.576
DD (mg/L)	0.98 (1.43)	0.98 (1.29)	0.987
ALT (U/L)	20.6 (14.5)	22.4 (17.4)	0.164
AST (U/L)	21.3 (12.8)	22.0 (13.6)	0.506
ALP (U/L)	85.7 (35.3)	83.5 (34.4)	0.451
GGT (U/L)	34.2 (32.1)	35.0 (31.1)	0.778
CHE (U/L)	7843 (2172)	7622 (2397)	0.248
TB (µmol/L)	10.4 (5.64)	10.8 (5.61)	0.443
DB (µmol/L)	3.66 (2.54)	3.80 (3.47)	0.563
IB (µmol/L)	6.74 (4.31)	6.97 (3.81)	0.524
TBA (µmol/L)	6.38 (5.45)	5.89 (5.43)	0.283
ALB (g/L)	40.2 (4.81)	39.8 (5.13)	0.434
GLU (mmol/L)	10.5 (4.24)	10.9 (4.59)	0.325
UA (µmol/L)	277 (98.7)	261 (90.9)	0.062
Cr (µmol/L)	65.8 (22.0)	63.7 (20.3)	0.241
BUN (mmol/L)	6.16 (2.47)	5.92 (2.52)	0.270
HCO3 (mmol/L)	23.4 (3.67)	23.4 (3.91)	0.978
TC (mmol/L)	4.48 (1.46)	4.48 (1.51)	0.976
TG (mmol/L)	1.99 (1.91)	1.93 (1.69)	0.689
HDL (mmol/L)	1.10 (0.39)	1.06 (0.35)	0.242
LDL (mmol/L)	2.55 (1.24)	2.52 (0.95)	0.775
ApoA (mmol/L)	1.03 (0.29)	1.00 (0.33)	0.281
ApoB (mmol/L)	0.82 (0.26)	0.82 (0.24)	0.885
CysC (mg/L)	1.14 (0.53)	1.04 (0.48)	0.026
RBP (mg/L)	36.4 (14.7)	34.4 (15.4)	0.119
SOD (KU/L)	153 (32.6)	150 (44.3)	0.318
TIR			0.442
TIR<70%	117 (59.7%)	258 (56.1%)	
TIR≥70%	79 (40.3%)	202 (43.9%)	
TAR (%)	28.4 (17.5)	28.1 (16.9)	0.800
TBR (%)	4.64 (7.04)	4.61 (6.77)	0.969
CV (%)	35.8 (8.17)	35.1 (7.42)	0.305
SD (mmol/L)	3.03 (0.85)	2.97 (0.84)	0.360
MAGE (mmol/L)	7.21 (2.07)	7.04 (2.15)	0.334
TyG	3.62 (0.76)	3.64 (0.78)	0.686
SHR	0.83 (0.32)	0.86 (0.36)	0.297
THR	2.19 (3.35)	2.07 (2.16)	0.639
Infection:			0.954
No	67 (34.2%)	160 (34.8%)	
Yes	129 (65.8%)	300 (65.2%)	

BMI, body mass index; SBP, systolic blood pressure; DBP, diastolic blood pressure; HbA1c, Glycated hemoglobin; WBC, white blood cell; NEU, neutrophil, LYM, lymphocyte; CRP, C-reactive protein; ALT, alanine aminotransferase; AST, aspartate aminotransferase; ALB, albumin; Cr, creatinine; TC, total serum cholesterol; TG, triglyceride; HDL, high-density lipoprotein; LDL, low-density lipoprotein; Glu, glucose; HbA1c, glycosylated hemoglobin; HGB, hemoglobin; D-D, D-dimer; GGT, γGlutamine Transferase; ALP, alkaline phosphatase; CHE, cholinesterase; TB, total bilirubin; DB, Direct bilirubin; IB, Indirect bilirubin; TBA, Total bile acids; UA, uric acid; BUN, Blood urea nitrogen; ApoA, Apolipoprotein A; ApoB, Apolipoprotein B; CysC, Cystatin C; RBP, Retinol binding protein; SOD, Superoxide Dismutase; TIR, time in range; TAR, time above range; TBR, time below range; CV, coefficient of variation of blood glucose; SD, blood glucose standard deviation; MAGE, mean amplitude of glycemic excursions; TyG, triglyceride-glucose; SHR, high-stress blood glucose ratio; THR, triglyceride/HDL ratio.

### 3.4 Using LASSO regression and the Boruta algorithm to screen predictive model variables in the training set

**Figure 2:**
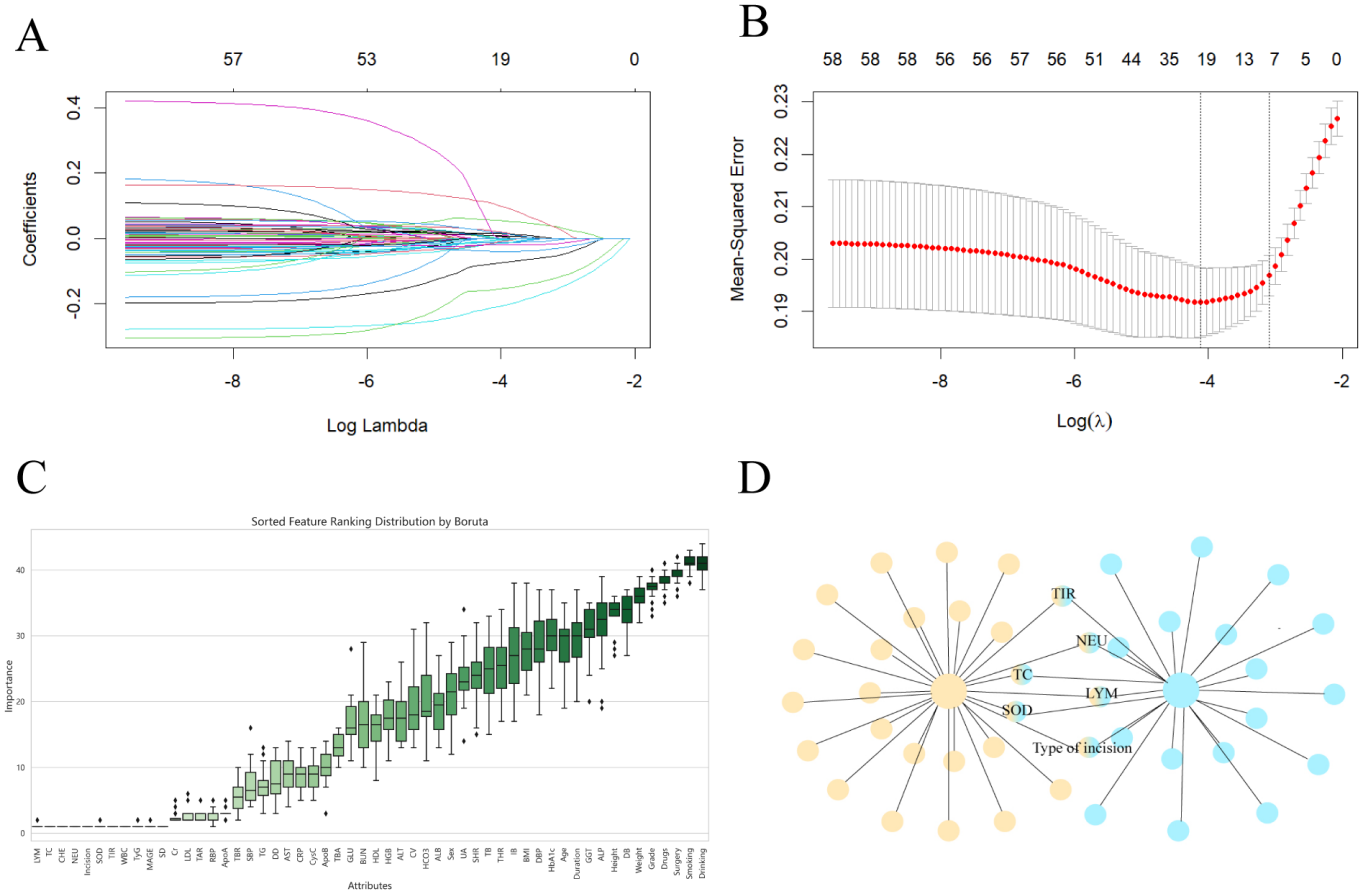
Screen predictive model variables in the training set. **(A)** LASSO regression pathway. Variable selection using LASSO logistic regression yields coefficient profiles for 56 variables. As the penalty coefficient λ increases, the coefficients of more and more variables are compressed until they are compressed to 0. **(B)** Cross-validation of LASSO regression. The best penalty coefficient lambda was selected using a twentyfold cross-validation and minimization criterion. The graph has log(lambda) in the horizontal coordinate, binomial deviance in the vertical coordinate, and vertical dashed lines plotted against one standard error criterion. Eight variables with nonzero coefficients were selected by optimal lambda. **(C)** Boruta. Identify the actual set of features by accurately estimating the importance of each feature. **(D)** The common subset of LASSO regression and Boruta.

LASSO regression, as a compressed estimation method, achieves variable selection and complexity adjustment by developing an optimization objective function that includes penalty terms (12). In this study, LASSO regression was used to identify feature factors and when λ=0.04524, 8 indicators, including sex, TIR, NEU, LYM, TC, SOD, surgical grade, and type of incision, were selected as feature variables (**Figure 2A**) and cross validated (**Figure 2B**). The Boruta algorithm is a feature selection and packaging algorithm based on random forests that evaluates the importance of features by generating “shadow variables” corresponding to each original variable in the dataset (13). The variables strictly related to postoperative infection selected by the Boruta algorithm are LYM, TC, CHE, NEU, SOD, TIR, WBC, TyG, MAGE, SD and type of incision (**Figure 2C**). By comparing and analyzing the screening results of the LASSO regression and Boruta algorithms, we decided to use a common subset of feature variables selected by the two methods, namely, TIR, LYM, NEU, TC, SOD, and the type of incision (**Figure 2D**), and ultimately used these six selected feature variables for model construction.

### 3.5 Establishing a nomogram model

**Figure 3:**
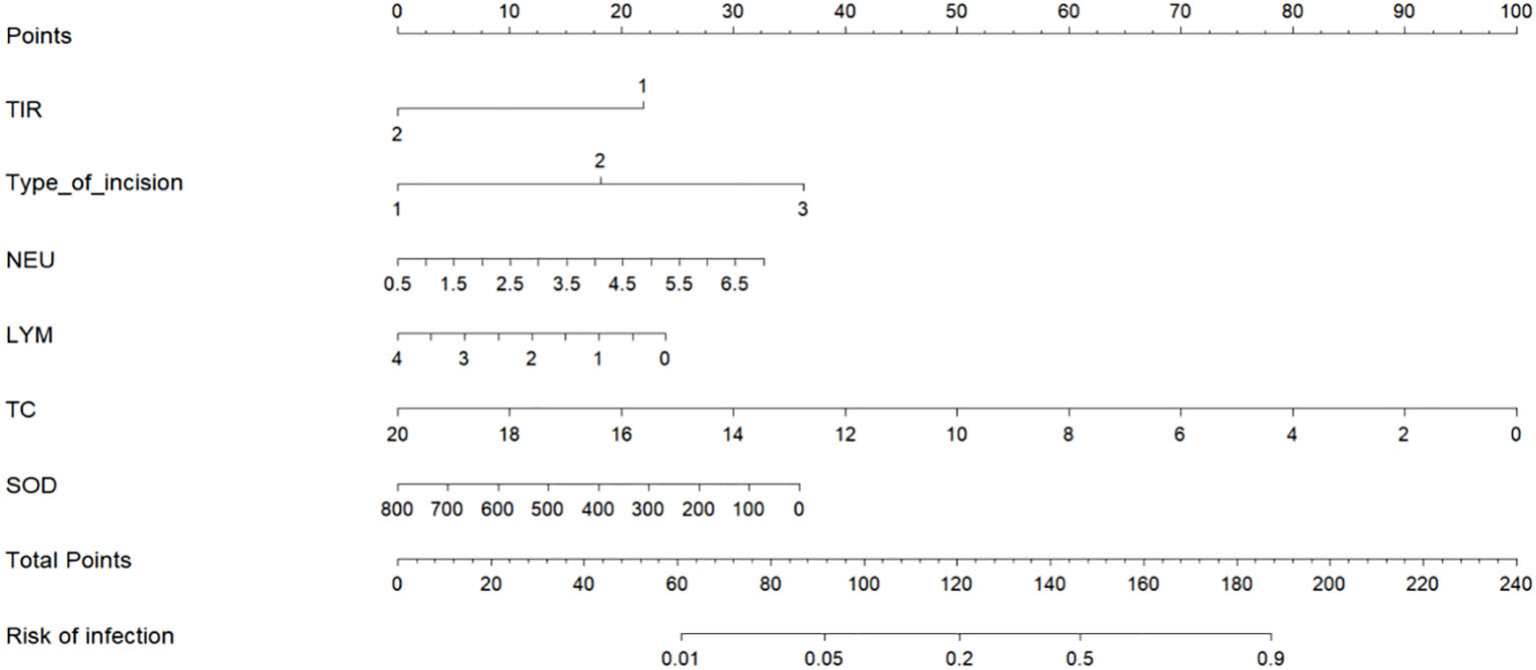
Nomogram of postoperative infection prediction model. The corresponding values of each variable are scored, and the total score is then obtained by summing the scores of all variables, and a vertical line plotted downward from the total score can be labeled to indicate the estimated probability of postoperative infection occurring in a patient with T2DM. TIR: 1: TIR<70% 2: TIR≥70%. Type of incision: 1: Type I incision 2: Type II incision 3: Type III incision.

We used the six characteristic variables (TIR, LYM, NEU, TC, SOD and type of incision) screened as predictors, established a nomogram, added the scores corresponding to each indicator level, and intuitively assessed the postoperative infection risk of patients with type 2 diabetes through the total score (**Figure 3**).

### 3.6 Validation of the nomogram for predicting postoperative infection risk in T2DM patients

**Figure 4:**
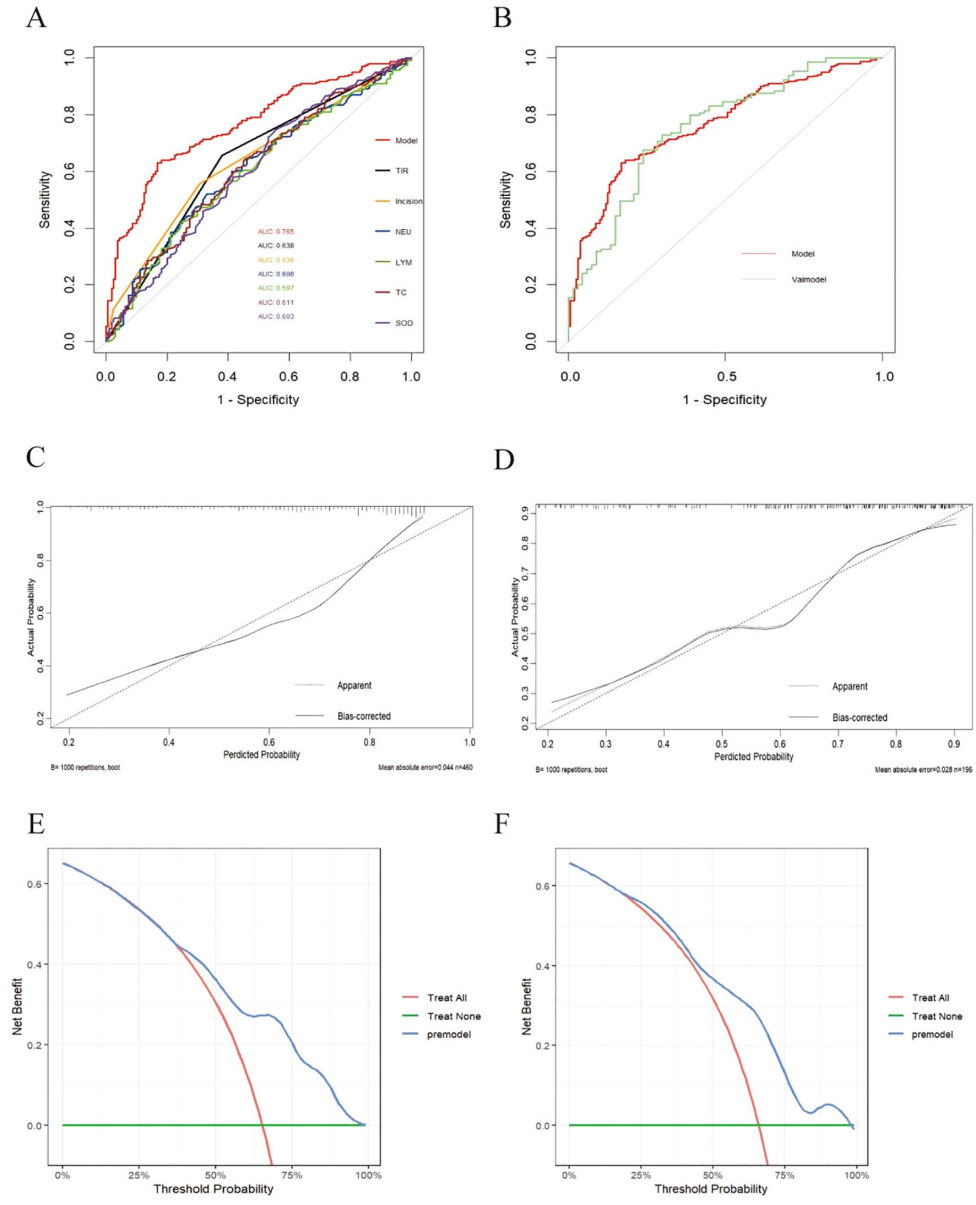
Validation of the nomogram. **(A)** Training set model and ROC curves of various indicators. **(B)** Comparison of ROC curves between training set (model) and validation set. **(C)** Calibration curve of the postoperative infection prediction model in the training set. The x-axis represents the predicted probability of the model. The y- axis represents the actual probability of occurrence. The diagonal dotted line represents a perfect prediction by an ideal model. The solid line represents the model curve calibrated by 1000 bootstrap resampling methods, a closer fit to the diagonal dotted line represents a better prediction. **(D)** Calibration curve of the postoperative infection prediction model in the testing set. **(E)** DCA curve of the postoperative infection prediction model in the training set. The x-axis in the figure represents the threshold probability, the y-axis represents the net benefit rate. The horizontal green solid line indicates that all patients did not receive clinical intervention, the red diagonal line indicates that all patients received clinical intervention, and the blue curve represents the net benefit rate of the prediction model. **(F)** DCA curve of the postoperative infection prediction model in the testing set.

ROC curve evaluation of predictive model performance revealed that the AUC of this predictive model was 0.765 (95% CI=0.721~0.809), and the predictive values of the individual predictive factors in this model were as follows: TIR and the type of incision predict the risk of postoperative infection in T2DM patients as 0.638 and 0.636, respectively, which are greater than those of TC (AUC=0.611), NEU (AUC=0.608), SOD (AUC=0.603), and LYM (AUC=0.597) (**Figure 4A**). Compared with a single predictive factor, the predictive value of using this model to predict postoperative infection risk in T2DM patients was greater. The area under the ROC curve of the validation set was 0.754 (95% CI=0.682–0.826), indicating good consistency between the training set and validation set (**Figure 4B**).

Calibration performance evaluation: As shown in **Figures 4C, D**, the prediction results of the model in the training set and validation set were relatively close to the actual situation.

DCA: As shown in the decision curve of the training set (**Figure 4E**), when the threshold probability was between 0.38 and 0.95, the net benefit provided by the model was significantly greater than that of the baseline decision. In the test set (**Figure 4F**), the model also showed good net gain, especially in the threshold probability range of 0.200.95, indicating that the model maintained a high level of net gain.

### 3.7 Subgroup forest plot results

**Figure 5:**
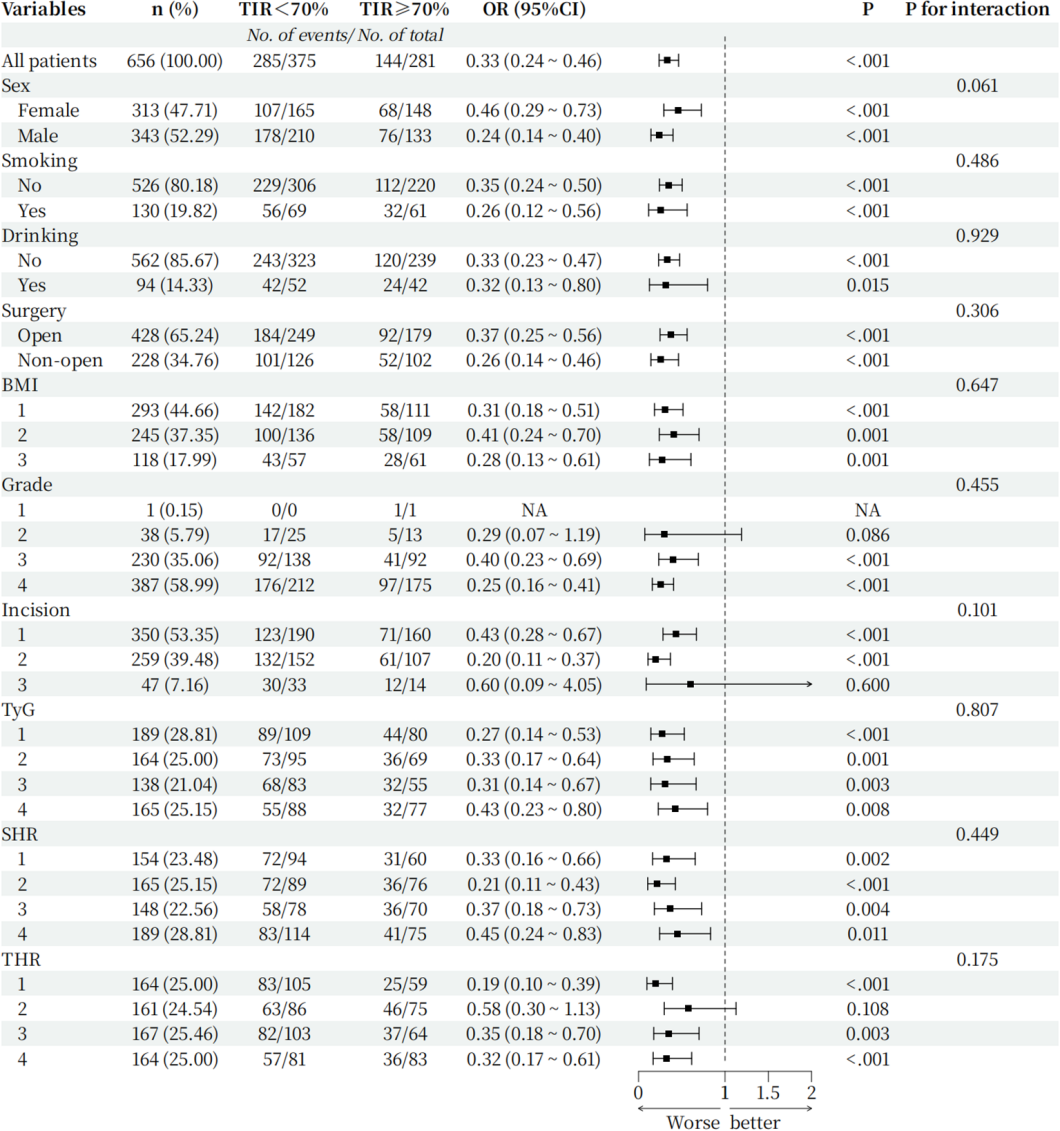
Hazard ratio for the primary outcome in prespecified subgroups. Incision grouping: 1: Type I incision 2: Type II incision 3: Type III incision; BMI grouping (Grouped by range criteria): 1: BMI<24 kg/m^2^ 2: 24kg/m^2^≤BMI<28 kg/m^2^ 3: BMI≥28 kg/m^2^; TyG grouping(Grouped by quartile): 1: TyG ≤ 3.20 2: 3.20<TyG ≤ 3.67 3: 3.67<TyG ≤ 4.07 4: TyG>4.07; SHR grouping(Grouped by quartile): 1: SHR ≤ 0.60 2: 0.60<SHR ≤ 0.80 3: 0.80<SHR ≤ 1.00 4: SHR>1.00; THR grouping (Grouped by quartile): 1: THR ≤ 0.97 2: 0.97<THR ≤ 1.50 3: 1.50<THR ≤ 2.37 4: THR>2.37.

To further verify that the TIR is robust among subgroups as a predictor of postoperative infection in T2DM patients, we conducted subgroup analysis, and the results are shown in **Figure 5**: sex, smoking status, alcohol consumption, BMI, surgical method, surgical grade, type of incision, TyG, SHR, and THR for subgroup analysis. The forest plot shows that when the TIR<70%, the proportion of postoperative infections was greater among different subgroups than when the TIR≥70%, and there was no significant interaction between the TIR and each subgroup(*p for interaction*>0.05).

## 4 Discussion

As of 2021, the International Diabetes Federation (IDF) estimates that the global population with diabetes has reached 537 million, accounting for 10.5% of the global population; by 2030, the prevalence of diabetes is expected to increase to 643 million (11.3%) (14). As the diabetic population continues to increase, the number of diabetic patients requiring surgical intervention will also increase. Studies have confirmed (15, 16) that poor blood glucose control is an independent risk factor for postoperative infections in various surgical operations. Postoperative infections often lead to a series of problems, such as poor wound healing, prolonged hospitalization, increased medical expenses, and even increased patient risk of death. The World Health Organization proposed that intensive perioperative hypoglycemia should be implemented in diabetic patients to reduce the risk of postoperative infection. However, patients undergoing strict blood glucose control may experience severe hypoglycemia, and the optimal blood glucose control range is still controversial (17). Therefore, finding a perioperative glucose-lowering target to reduce postoperative infections is crucial. CGM is a continuous blood glucose monitoring technology that changes the blood glucose monitoring method from blood to interstitial fluid and integrates and divides all monitored blood glucose values into TIR, TAR and TBR. TIR has become a popular choice for clinicians and patients because it can more intuitively express blood glucose control levels.

The modified Poisson model is a statistical method that can calculate the relative risk on the basis of robust error variance, which is more accurate than the commonly used logistic regression results. According to *the International Consensus on Time in Range* (8), we recommended a TIR of 70% as the cutoff point to study perioperative T2DM patients. Through modified Poisson regression analysis, we found that the TIR is a risk factor for postoperative infection in T2DM patients, and when the TIR is <70%, the risk of postoperative infection in T2DM patients increases by 52.2%. Thus, for perioperative T2DM patients, poor preoperative TIR control leads to an increased risk of postoperative infection, and blood sugar should be strictly controlled before surgery.

HbA1c, which is used as a standard to evaluate blood sugar control levels, is often affected by multiple factors and fails to reflect blood sugar fluctuations, which are related to diabetic complications (18, 19). In 2017, Jourdan et al. reported that HbA1c was related to postoperative deep infection in T2DM patients who underwent total hip arthroplasty (TKA), but HbA1c could not be used as an independent risk factor for deep infection after TKA (20). In contrast to HbA1c, the TIR can better reflect recent blood sugar fluctuations in real time, and the TIR is better than HbA1c in terms of the operability of blood sugar monitoring and the prediction of diabetic complications (21). As one of the manifestations of blood glucose fluctuation amplitude, the higher the TIR value, the smaller the blood glucose fluctuation amplitude, indicating that the blood glucose is in a relatively stable state (22). When blood glucose fluctuations are too large, the antioxidant capacity of the cells themselves decreases, which may aggravate cellular oxidative stress, leading to damage to endothelial cell function and the induction of inflammatory reactions (23). Experimental studies have shown that prolonged hyperglycemia or excessive fluctuations in blood sugar can reduce the immune function of cells at the site of infection (24). Liu et al., in their study on postoperative infection after lumbar fusion in patients with T2DM, reported that the amplitude of preoperative blood glucose fluctuations was closely related to postoperative infection and could be used as an independent risk predictor of postoperative infection after this surgery (25). TIR is negatively correlated with the amplitude of blood glucose fluctuations. We can use CGM to monitor blood sugar and increase the percentage of TIR to reduce the amplitude of blood glucose fluctuations (26), thereby reducing the risk of postoperative infection. This is consistent with the fact that strict control of blood sugar can reduce the risk of postoperative infection. This finding is consistent with the view of postinfection risk (27).

Although the TIR is an influencing factor for postoperative infection in T2DM patients, can the TIR be used to predict the risk of postoperative infection in T2DM patients? To this end, we established a clinical prediction model that can identify potential correlations between preoperative indicators and postoperative infection outcomes, allowing clinicians to make more accurate predictions of the risk of postoperative infection in T2DM patients so that relevant measures can be taken in a timely manner to reduce this risk. In this study, we used two algorithms to screen variables, among which Lasso regression stands out in data analysis and collinearity processing through automatic variable selection and sparse modeling, while Boruta algorithm performs more outstandingly in nonlinear relationships, with strong stability and can directly provide feature importance ranking and classification results. To eliminate the collinearity of the model, we used the dual method of LASSO regression and the Boruta algorithm to determine the accuracy of the feature variables and the stability of the prediction model (28). Finally, both LASSO regression and the Boruta algorithm revealed that the TIR was included in the clinical prediction model as a characteristic variable of postoperative infection in T2DM patients. To increase the readability of the model and help clinicians obtain relevant risk probabilities through simple calculations, we presented all characteristic variables in the form of nomograms.

In the clinical prediction model, in addition to TIR, we included NEU, LYM, TC, SOD and surgery type. HbA1c was not included in part because anemia and hypoalbuminemia affect the accuracy of HbA1c in some patients with tumors or pregnant caesarean sections, and the inability of HbA1c to respond to blood sugar fluctuations in a timely manner is also a major drawback. WBC, NEU, LYM, and CRP are commonly used inflammatory markers in clinical practice, and are more easily obtained from routine examinations compared to inflammatory factors such as tumor necrosis factor or interleukin. But studies have shown that CRP has a weak correlation with metabolic diseases and is easily affected by HDL (29). WBCs have poor sensitivity and specificity, and NEU is at the forefront of the body’s defense against pathogenic invasion and can first reach the site of inflammation when infection occurs (30). LYM plays a core role in the body’s immune response and can respond to viral infections in a timely manner. Therefore, jointly incorporating NEU and the LYM into the prediction model is better than incorporating a single WBC indicator. TC is the major steroidal compound in mammals and may trigger inflammatory cascades when regulating intracellular and intracellular homeostasis (31). Morimoto et al. also confirmed in a study on total serum cholesterol and postoperative internal infections of the gastrointestinal tract (32) that there was a reverse-J-type correlation between total serum cholesterol and postoperative internal infections, which may be due to the ability of circulating cholesterol rich lipoproteins and triglyceride rich lipoproteins to bind and detoxify bacterial lipopolysaccharides. Thus, TC was used as a reliable predictor of postoperative infection. SOD is an antioxidant metalloenzyme that exists in organisms. It plays a vital role in oxidative stress in the body and is inseparable from infection. It is associated with early death in acute pancreatitis and stroke infection (33, 34). In a mouse experiment by Christina et al. (35), SOD also showed a protective effect during lung infection. This is consistent with the fact that, in this study, the risk of postoperative infection gradually increased as SOD levels decreased. The results are consistent. The Centers for Disease Control and Prevention classify surgical incisions into 4 categories: Type I/clean incision, Type II/clean-contaminated incision, Type III/contaminated incision, and Type IV/infected incision (36). Studies have shown that the type of surgical incision is related to postoperative infection (10). Our study also revealed that, as the level of incision type increases, the risk of postoperative infection increases, which suggests that we can take corresponding preventive measures according to the different types of postoperative incisions used in T2DM patients.

In this study, we also conducted a series of subgroup analyses, and the results suggested that the TIR is robust among subgroups and is an independent prognostic factor for postoperative infection in patients with T2DM. Notably, this study retained some Class III incisions that met the inclusion criteria. Subgroup analysis revealed that, when the surgical incision type was a Class III incision, even if the TIR was ≥70%, more people would develop postoperative infections. This finding suggests that when the postoperative incision is contaminated, although preoperative blood sugar control is acceptable, the risk of postoperative infection is still high. We should take relevant preventive measures in a timely manner after surgery to reduce the risk of postoperative infection.

In recent years, composite indicators such as TyG, SHR, and THR have been shown to be related to the risk of cardiovascular and cerebrovascular diseases, sepsis, and postoperative gastrointestinal infections (37–39). Therefore, this study also included these new indicators. The composite indicators were analyzed, but the results suggested that these indicators were not related to postoperative infection in patients with T2DM. This may be because these indicators are strongly related to single-disciplinary surgeries, and there were many types of surgeries in our study that were not limited to gastrointestinal surgery, tract surgery or cardiac surgery and therefore were not suitable for this study.

## 5 Conclusion

In conclusion, our study shows that the TIR is not only a risk factor for postoperative infection in patients with perioperative T2DM but also has good predictive value for postoperative infection. When the preoperative TIR is lower than 70%, TIR is less likely to cause postoperative infection in T2DM patients. The risk will be greatly increased. In most previous studies, prediction models have been used only to predict diabetic complications or surgical outcomes, and few studies have investigated postoperative infection in patients with T2DM. This model is more helpful for clinicians to evaluate conditions on the basis of individual patient conditions, reduce the occurrence of postoperative infections and shorten the length of hospitalization. However, this study also has shortcomings. This was a single-center study that lacked external verification, only observed the occurrence of infections during hospitalization, and cannot be used to evaluate the situation after discharge. Therefore, the next step is to evaluate the long-term predictive value of the model after surgery, and the accuracy of the model will be further tested through external validation.

## Data Availability

The original contributions presented in the study are included in the article/**Supplementary Material**. Further inquiries can be directed to the corresponding author.
