# Supplementary material for: Assessing the predictive value of time-in-range level for the risk of postoperative infection in patients with type 2 diabetes: a cohort study
Source: Front Endocrinol (Lausanne). 2025 Apr 15;16:1539039. doi: 10.3389/fendo.2025.1539039 (PMC12037399; doi:10.3389/fendo.2025.1539039)
Supplement: Supplementary file 1 [file Table1.docx]

S1. Analysis of Interaction between Subgroups

| Subgroups | B | *p* | Exp(B) | 95%*CI* | |
| --- | --- | --- | --- | --- | --- |
|  |  |  |  | lower limit | upper limit |
| Sex by TIR | 0.653 | 0.061 | 1.922 | 0.971 | 3.805 |
| Smoking by TIR | -0.308 | 0.486 | 0.735 | 0.308 | 1.750 |
| Drinking by TIR | -0.045 | 0.929 | 0.956 | 0.356 | 2.570 |
| Surgery by TIR | -0.372 | 0.306 | 0.689 | 0.338 | 1.406 |
| BMI by TIR | 0.002 | 0.647 | 1.002 | 0.642 | 1.566 |
| Surgical grade by TIR | -0.292 | 0.455 | 0.747 | 0.434 | 1.285 |
| Incision scoring by TIR | -0.448 | 0.101 | 0.639 | 0.343 | 1.192 |
| TyG by TIR | 0.127 | 0.807 | 1.136 | 0.849 | 1.520 |
| SHR by TIR | 0.140 | 0.449 | 1.151 | 0.858 | 1.544 |
| THR by TIR | 0.060 | 0.175 | 1.062 | 0.788 | 1.432 |
